# Community-acquired Pneumonia Secondary to Legionella pneumophila and Streptococcus pneumoniae: A Rare Co-infection

**DOI:** 10.7759/cureus.4080

**Published:** 2019-02-16

**Authors:** Moeezullah Beg, Hamza Arif, Thomas Walsh

**Affiliations:** 1 Internal Medicine, University of Texas Health Science Center San Antonio, San Antonio, USA; 2 Internal Medicine, Allegheny Health Network, Pittsburgh, USA; 3 Infectious Disease, Allegheny Health Network, Pittsburgh, USA

**Keywords:** community-acquired pneumonia, streptococcus pneumonia, legionella pneumophilia, co-infection, urinary antigens

## Abstract

Community-acquired pneumonia (CAP) is a frequent cause of hospitalization in adults. S*treptococcus pneumoniae* is the most commonly identified pathogen in CAP whereas *Legionella pneumophilia* is infrequently identified in CAP. Although co-infections have been previously described, the presence of both pneumococcus and legionella together is rare. We present a patient with positive urinary antigens for both *Streptococcus pneumoniae* and *Legionella pneumophilia* serogroup 1, indicating an unusual co-infection.

## Introduction

Community-acquired pneumonia (CAP) is a frequent cause of hospitalization in adults leading to significant morbidity and mortality [1]. Most cases of bacterial CAP are caused by a single pathogen, with *Streptococcus pneumoniae *(*S. pneumoniae*) identified most commonly [2]. Alternatively, *Legionella pneumophila *(*L. pneumophila*) is a less common cause of CAP [2-5]. Although co-infections of various bacterial and viral microorganisms have been described, the presence of both *S. pneumoniae* and *L. pneumophila* together in CAP is rare [2, 6]. We describe a patient with CAP diagnosed with positive urinary antigens for both *S. pneumoniae* and *L. pneumophila* serogroup 1 (Lp1), indicating an unusual co-infection. A part of this article was presented at the American College of Chest Physicians' annual meeting, CHEST 2017, in Toronto, Canada.

## Case presentation

An 80-year-old Caucasian female who was an active smoker with a 40 pack year smoking history and a past medical history of primary hypertension and chronic obstructive pulmonary disease (COPD) presented to our hospital emergency department (ED) in February, 2017 with fatigue, generalized weakness, and shortness of breath for the last two days. She had been noticing progressively worsening shortness of breath on exertion without any fever, chills, cough, chest pain, orthopnea or paroxysmal nocturnal dyspnea. She did not have any sick contacts and had not travelled anywhere recently.

Upon arrival to the hospital, she was noted to have a heart rate of 122 beats per minute, a respiratory rate of 30 breaths per minute with an oxygen saturation of 85% on room air, and a blood pressure of 161/86 mmHg. Further physical examination revealed a thin and cachectic female who appeared to be in mild respiratory distress. She was noted to have normal heart sounds without any murmurs, rubs, or gallops. A pulmonary examination revealed expiratory wheezing bilaterally without any rales or rhonchi. She did not have rashes or peripheral edema. Due to her respiratory distress she was started on non-invasive ventilatory support in the emergency department (ED), which led to improvement in her respiratory status.

**Figure 1:**
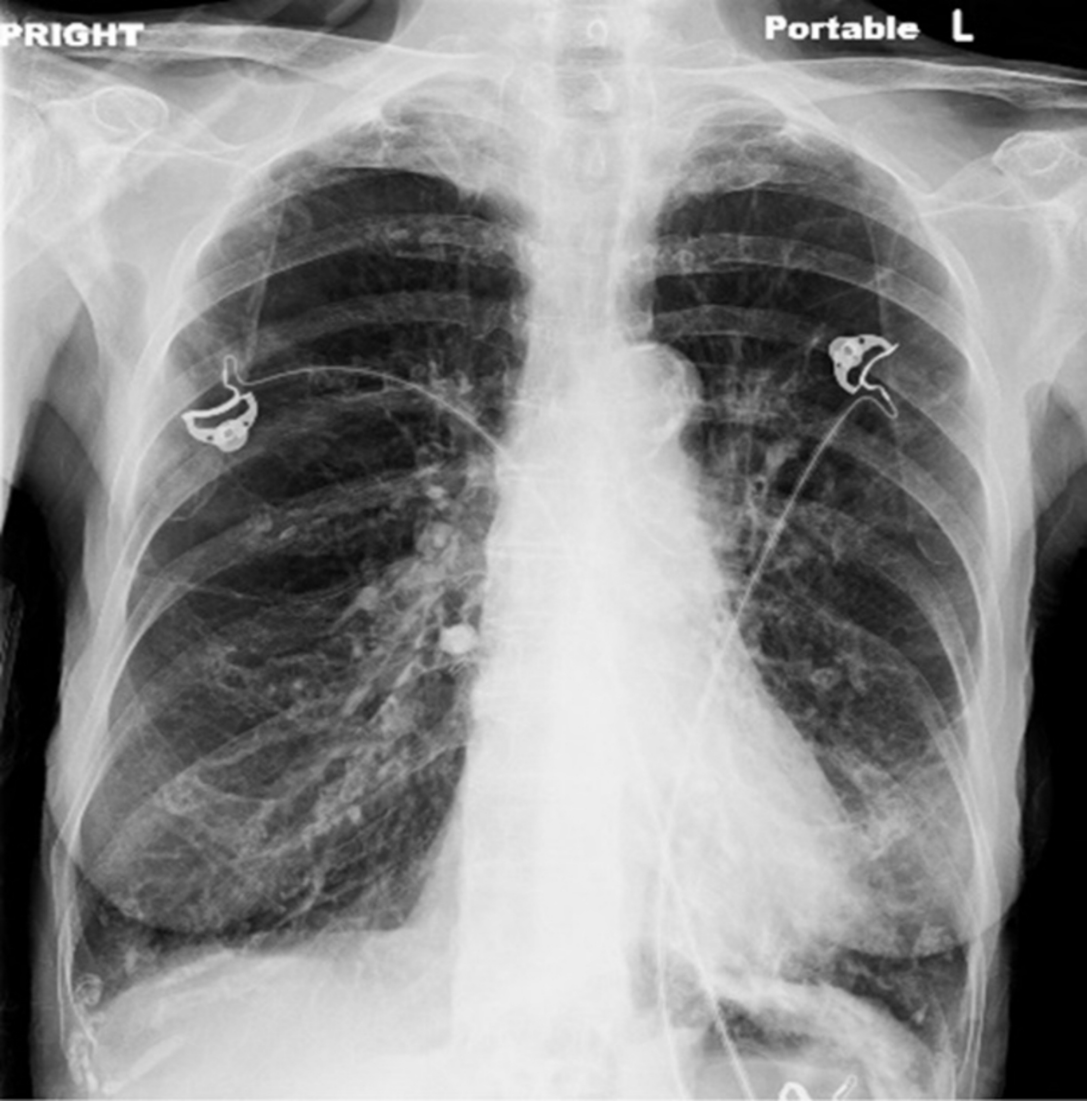
Chest X-ray revealing hyper-inflated lungs without any focal consolidation.

Laboratory investigation revealed a hemoglobin count of 15.1 g/dL (reference range [ref], 12.3-15.3), a peripheral white blood cell count of 21,130 cells/mm3 (ref, 4400-11,300) with a relative neutrophil percentage of 80% (ref, 37%-77%), a platelet count of 301,000/mm3 (ref, 145,000-445,000), a sodium level of 134 mmol/L, a serum creatinine level of 0.30 mg/dL (ref, 0.70-1.5), and a blood urea nitrogen of 8 mg/dL (ref, 9-20). Her liver enzymes were within normal limits. Venous blood gas analysis revealed a pH of 7.26 and a CO2 of 83 mmHg (ref, 41-51). Initial serum procalcitonin was 0.07 ng/mL and a chest radiograph was only significant for hyper-inflated lungs (Figure 1).

**Figure 2:**
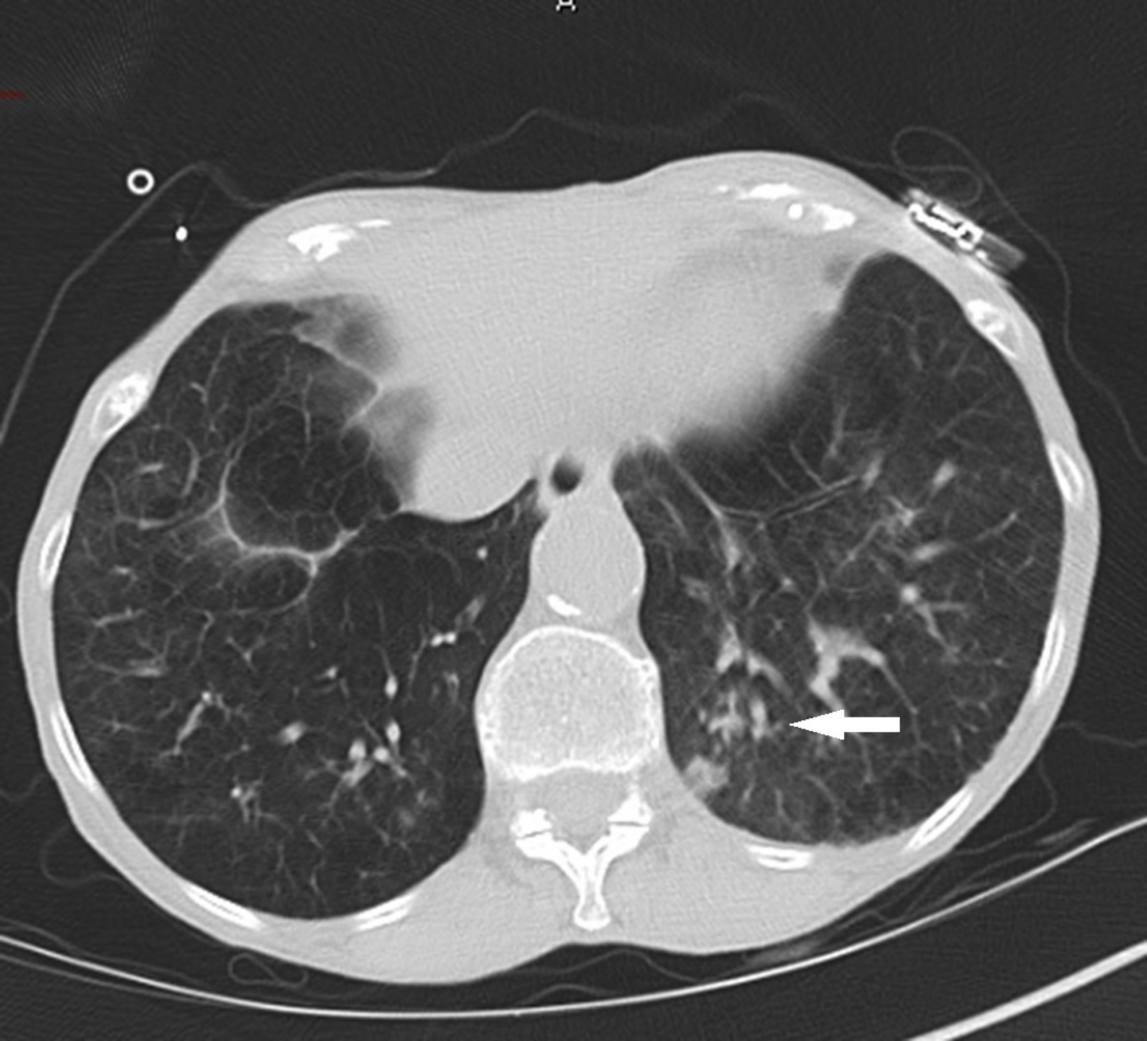
Computed tomography of the chest showed tree-in-bud opacities along with bronchial wall thickening (arrow) consistent with an infectious process.

She was admitted to the hospital for management of mild COPD exacerbation and treated with inhaled bronchodilators and systemic steroids. Repeat blood work obtained 12 hours after initial presentation was significant for a serum procalcitonin of 2.07 ng/mL and a peripheral white blood cell count of 35,170 cells/mm3 with a relative neutrophil percentage of 91% and 2% bands. Due to concerns for development of CAP, sputum cultures, two sets of blood cultures via peripheral blood draw and urinary antigens for *S. pneumoniae* and *L. pneumophila* were obtained. She was started on intravenous ampicillin-sulbactam 3 grams every six hours and azithromycin 500 milligrams every 24 hours. A computed tomography scan of the chest revealed tree-in-bud opacities along with regions of bronchial wall thickening in the bilateral lower lobes (Figure 2).

A sputum Gram stain revealed many Gram-positive rods, rare Gram-positive cocci, less than 10 squamous epithelial cells and greater than 25 white blood cells per low power field. Her blood cultures remained negative and the final sputum culture grew normal respiratory flora. Surprisingly, urinary antigens for both *S. pneumoniae* and Lp1 returned positive. Antibiotics were then de-escalated to intravenous levofloxacin 500 mg every 24 hours for a total of five days. The patient’s clinical status improved, and she was discharged home in a stable condition after a one-week hospital stay.

## Discussion

Although co-infections with *S. pneumoniae* and *L. pneumophila* have been described, this appears to be only the second case where a CAP co-infection was diagnosed on the basis of positive urinary antigens [4]. Various studies have shown high specificity and positive predictive value (PPV) of urinary antigens. Thus, our case most likely represents a true co-infection leading to CAP.

One study revealed a specificity of 96%, a PPV of 95.1%, and a likelihood ratio of 19.9 for pneumococcal urinary antigen [7]. Similarly, urinary antigen testing for legionella is also highly specific (99.1%-99.5%) indicating that a positive result in the appropriate clinical setting represents a true infection [8-10]. A systematic analysis of 30 studies revealed a pooled specificity of 99.1% for legionella urinary antigen [10]. However, legionella urinary antigen only detects Lp1 and thus sputum culture on special media remains the gold standard for diagnosis of legionella infections.

Urinary antigen testing should be routinely utilized to identify etiological agents of CAP due to their excellent specificity, but given the poor sensitivities of these tests, a negative result should not be used to rule out infection caused by *S. pneumoniae* or *L. pneumophila*.

False positive cases have been rarely reported for legionella urinary antigen. One case had exposure to rabbit serum antibodies and the other patient had pneumonia and a brain lesion from *Nocardia asteroides* [11, 12]. In our patient, although a false positive result is a possibility as the sputum culture for legionella was not performed, she likely had true CAP with legionella and pneumococcus due to her classic signs and symptoms, high specificity of urinary antigen testing, as well as rapid response to antibiotics.

## Conclusions

This unique case of *S. pneumoniae* and *L. pneumophila* co-infection demonstrates the importance of urinary antigen testing in patients hospitalized secondary to CAP in addition to obtaining sputum cultures and peripheral blood cultures. This approach will promote early institution of organism-specific antibiotic therapy and foster antibiotic stewardship.
